# Omicron variant of SARS-CoV-2 exhibits an increased resilience to the antiviral type I interferon response

**DOI:** 10.1093/pnasnexus/pgac067

**Published:** 2022-05-23

**Authors:** Lyudmila Shalamova, Ulrike Felgenhauer, Jochen Wilhelm, Andreas R Schaubmar, Kathrin Büttner, Andreas Schoen, Marek Widera, Sandra Ciesek, Friedemann Weber

**Affiliations:** Institute for Virology, FB10-Veterinary Medicine, Justus-Liebig University, D-35392 Giessen, Germany; Institute for Virology, FB10-Veterinary Medicine, Justus-Liebig University, D-35392 Giessen, Germany; Institute for Lung Health (ILH), Justus-Liebig-University Giessen , D-35392 Giessen, Germany; Unit for Biomathematics and Data processing, FB10-Veterinary Medicine, Justus-Liebig University, D-35392 Giessen, Germany; Unit for Biomathematics and Data processing, FB10-Veterinary Medicine, Justus-Liebig University, D-35392 Giessen, Germany; Institute for Virology, FB10-Veterinary Medicine, Justus-Liebig University, D-35392 Giessen, Germany; Institute for Medical Virology, University Hospital, Goethe University, D-60596 Frankfurt am Main, Germany; Institute for Medical Virology, University Hospital, Goethe University, D-60596 Frankfurt am Main, Germany; German Centre for Infection Research (DZIF), D-35392 Giessen and D-60596 Frankfurt, Germany; Branch Translational Medicine and Pharmacology, Fraunhofer Institute for Molecular Biology and Applied Ecology (IME), 60596 Frankfurt am Main, Germany; Institute for Virology, FB10-Veterinary Medicine, Justus-Liebig University, D-35392 Giessen, Germany; German Centre for Infection Research (DZIF), D-35392 Giessen and D-60596 Frankfurt, Germany

**Keywords:** COVID-19, SARS-CoV-2, Omicron variant BA.1, interferon induction, interferon sensitivity

## Abstract

The new variant of concern (VOC) of SARS-CoV-2, Omicron (B.1.1.529), is genetically very different from other VOCs. We compared Omicron with the preceding VOC Delta (B.1.617.2) and the wildtype (wt) strain (B.1) with respect to their interactions with the antiviral interferon (IFN-alpha/beta) response in infected cells. Our data indicate that IFN induction by Omicron is low and comparable to the wt, whereas Delta showed an increased IFN induction. However, Omicron exceeded both the wt and the Delta strain with respect to the ability to withstand the antiviral state imposed by IFN-alpha.

## Introduction

Omicron is a new variant of concern (VOC) of the pandemic SARS-CoV-2 causing COVID-19. Since its detection in South Africa in November 2021 (1), Omicron has rapidly spread in all countries where it was introduced, indicating elevated infectivity and a certain resistance to pre-existing immunity (2). These features are due to an unprecedented number of mutations that are distinguishing Omicron from the original coronavirus that emerged end of 2019, as well as from the subsequently appearing VOCs like e.g. Delta (1).

Type I interferons (IFN-alpha/beta) constitute the first innate immune response to invading viruses (3). Viral RNA structures are recognized by cellular sensors that trigger the induction of IFN genes. Secreted IFN binds to its cognate receptor and stimulates expression of antiviral genes. SARS-CoV-2, therefore, evolved a series of countermeasures to the IFN-stimulated antiviral state (4). Nonetheless, SARS-CoV-2 still induces a certain level of type I IFNs and other cytokines (5) and exogenously added IFN is inhibitory to viral replication (5–7).

## Results

**Fig. 1.**
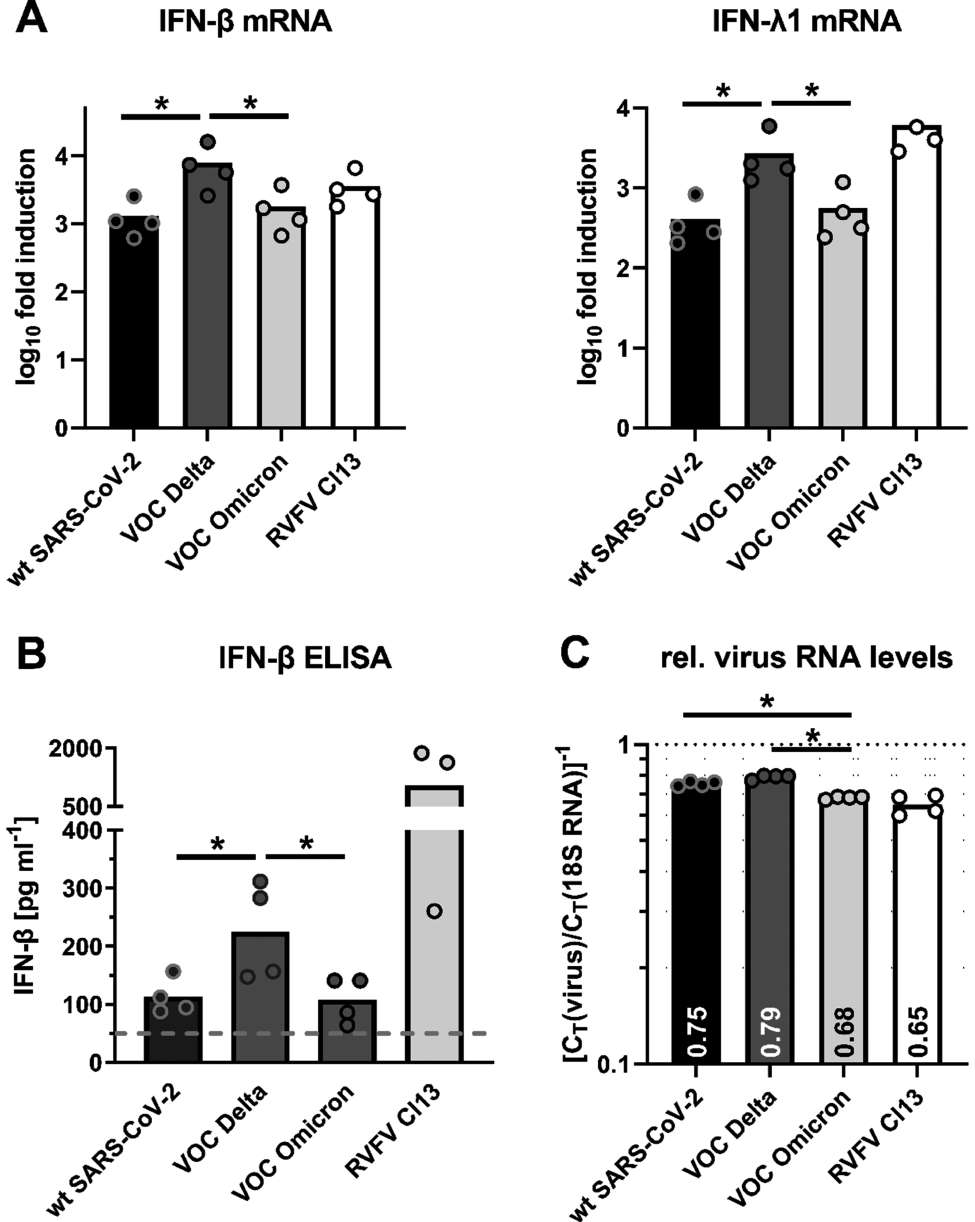
IFN induction by strains of SARS-CoV-2. Calu-3 cells were infected for 24 h (MOI 1), and analyzed by RT-qPCR for IFN mRNAs (A). Shown are fold-induction over the uninfected mock control, normalized to 18S rRNA. (B) IFN-β in supernatants. Dashed line: detection limit. (C) Viral RNA presented as C_T_ values for E (coronaviruses) or L (RVFV Cl13) genes normalized to 18S rRNA. Data points (dots) and mean values (bars) are shown. The log_10_ transformed values of the different coronaviruses were pairwise tested by one-factorial ANOVA with Tukey correction for multiple comparisons. * indicates *P* < 0.05. All other coronavirus comparisons had *P-*values > 0.05.

We investigated interactions of Omicron with the IFN system, in comparison to the parental “wt” strain and the preceding VOC, Delta. As a first step, IFN induction was measured in the Calu-3 human lung cell model using RT-qPCR for mRNAs for type I IFN-beta and type III IFN-lambda1, and by ELISA for secreted IFN-beta. As positive control, we used the IFN-inducing Rift Valley fever virus (RVFV) mutant Clone 13 (8). Figures 1(A) and (B) show that all SARS-CoV-2 strains induced IFN, but that Omicron and wildtype (wt) were weaker than Delta. Levels of viral RNA, however, were similar for wt and Delta, but lower for Omicron (Fig. 1C). Therefore, whereas Delta seems to have a diminished IFN-antagonistic activity compared to wt, Omicron may be a weaker inducer because it generates less IFN-inducing RNAs.

**Fig. 2.**
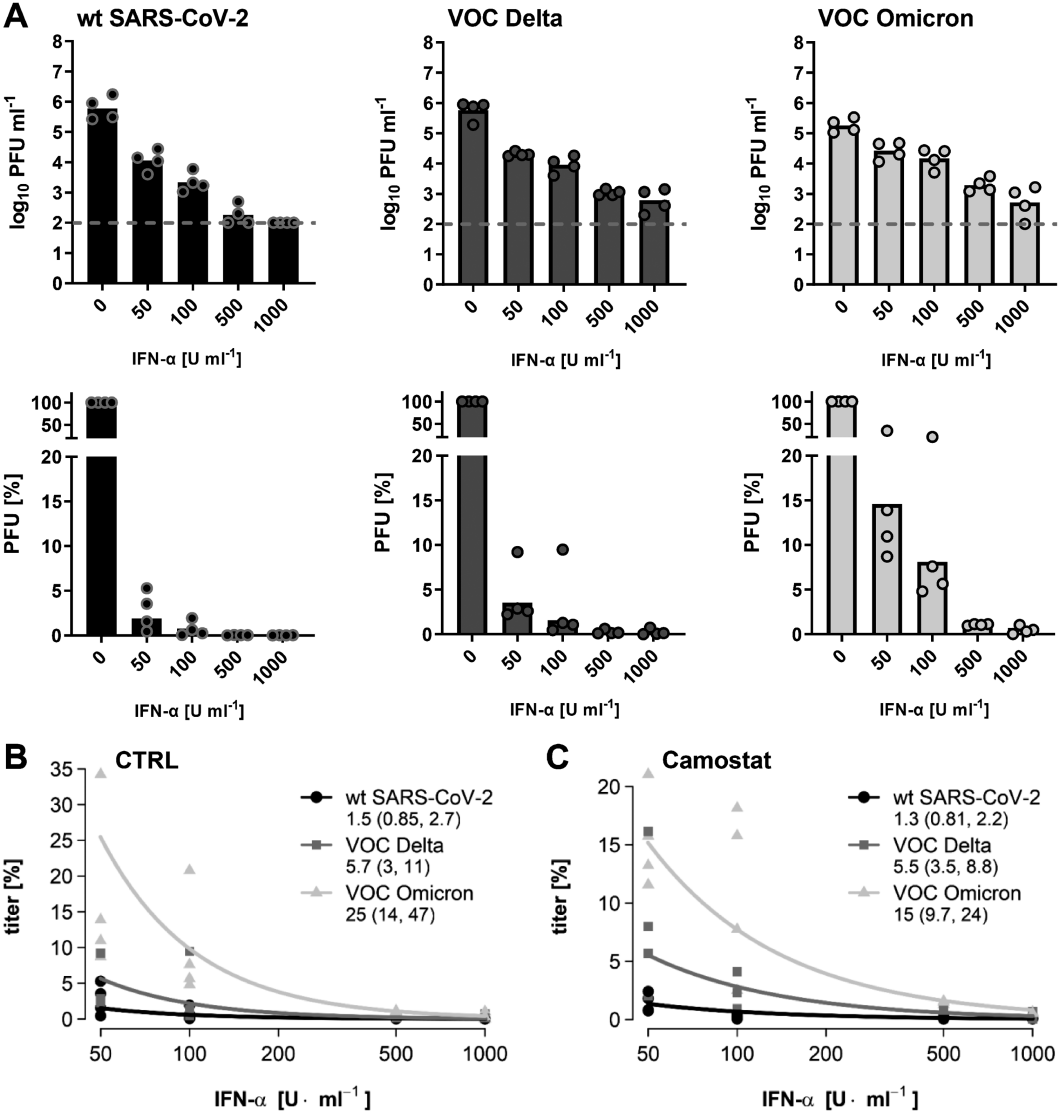
Sensitivity to type I IFN dose escalation. (A) Calu-3 cells were treated with increasing amounts of IFN-alpha prior to infection at an MOI of 0.01 for 24 h. Upper panel: titers and lower panel: data normalized to the nontreated control. Titer values below the detection level (dashed line) were set to 100 PFU/ml. (B) Regression analysis of the dose–response data shown in (A). Numbers underneath the virus names are estimators for the titers at 50 U/ml IFN, confidence intervals are given in brackets. All comparisons had *P-*values < 0.05. (C) Analysis of IFN dose–response curves in cells that were additionally pretreated with 1 µM Camostat. Note that for reasons of compatibility, pretreatment in (A) contained DMSO.

To investigate the other end of the IFN response, namely sensitivity to antiviral action, we pretreated cells with increasing amounts of IFN-alpha, infected them with the strains, and measured virus yields 24 h later. Figure 2(A) shows that IFN reduces all viruses in a dose-dependent manner. However, 50 units IFN/ml suppressed Omicron to about 15%, but wt and Delta to below 10%. The dose–response curves were analyzed by a generalized linear mixed model that included a fixed intercept term for each variant and a common slope coefficient, so that the intercepts code the expected titer at 50 U/ml IFN. All slopes intercepts were statistically different from each other, with Omicron being the least affected by IFN, followed by Delta and wt being the most affected (Fig. 2B). SARS-coronaviruses can enter cells either at the plasma membrane or by endosomal entry, but Omicron has evolved toward the latter mode (9). To see whether these differences could influence IFN sensitivity, we disabled the plasma membrane entry route by applying the TMPRSS2 inhibitor Camostat. Nonetheless, the intercepts of the IFN dose–response curves were still statistically different for the three viruses, again with Omicron being the least affected (Fig. 2C). Apparently, the mode of virus entry is not determining IFN sensitivity differences.

**Fig. 3.**
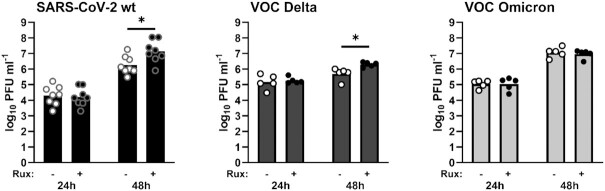
Effect of the IFN signaling inhibitor Ruxolitinib (Rux) on virus replication. Calu-3 cells were pretreated with Ruxolitinib for 24 h, infected at MOI 0.001 (wt) or 0.01 (VOC Delta and Omicron), and supernatants titrated at the indicated time points. Log-transformed titers were analyzed by unpaired two-tailed t tests. Asterisk * indicates *P* < 0.05. All other comparisons had *P-*values > 0.05.

All viruses including Omicron were inducing IFN (see Fig. 1). To measure a potential influence on virus replication, we incapacitated the IFN system with Ruxolitinib. This treatment could improve titers of wt and Delta, but not of Omicron (Fig. 3). Thus, whereas wt and Delta benefit from artificial IFN suppression, Omicron is independent of it.

## Discussion

IFNs were shown to restrict wt SARS-CoV-2 in cell culture (5–7), and in patients an early IFN therapy is associated with reduced mortality (10). This may also apply to the IFN that is endogenously produced by the infected individual. In children, a preactivated IFN system is controlling infection (11), whereas in the elderly the IFN system is less active and can be additionally hampered by anti-IFN autoantibodies (12). Thus, virus-IFN system interactions are a determinant of COVID-19 risk, and the outcome of infection depends on the timing and amount of IFN production and on the degree of viral resistance. Earlier reports have shown that the more pathogenic SARS-CoV-1 is more IFN-resistant than wt SARS-CoV-2 (6, 7). Therefore, IFN resilience, most likely enabled by the hallmark mutations in the IFN antagonistic proteins (13), is an additional feature besides immune escape, increased ACE2 affinity and endosomal entry (9) and may contribute to the hypertransmissibility of Omicron.

## Materials and Methods

### Cells and viruses

Calu-3, VeroE6, and VeroE6-TMPRSS2 cells (kindly provided by Stefan Poehlmann) were cultivated in DMEM with 10% FBS. SARS-CoV-2 strains “wt” (München-1.2/2020/984 (B.1) (14); kindly provided by Christian Drosten), “VOC Delta” (B.1.617.2 (FFM-IND8424/2021) (15)), and “VOC Omicron” (B.1.1.529 (FFM-SIM0550/2021) (16)) were grown on VeroE6-TMPRSS2 cells (wt) or VeroE6 cells (VOCs), and titrated on VeroE6. Infections were done under BSL3 conditions.

### Interferon induction

Calu-3 cells seeded into 24-well plates were infected at a multiplicity of infection (MOI) of 1. At 24 h postinfection, RNAs were extracted (RNeasy, Qiagen), and reverse transcribed (prime Script RT reagent, Takara). Cellular and viral cDNAs were measured using TB Green Premix Ex Taq II (Tli RNase H Plus, Takara) and Premix Ex Taq (probe qPCR, Takara), respectively. Primers and probes were described earlier (8, 14). Supernatants were measured using the IFN beta Human ELISA Kit (Invitrogen).

### Inhibitor and interferon treatment

Cells seeded into 24-well plates were pretreated for 24 h with pan-species IFN-alpha (B/D; PBL Assay Science) or 1 µM Ruxolitinib (Selleckchem), or for 2 h with 1 µM Camostat (Selleckchem) or DMSO, all also added to inoculum and the incubation medium. Infections were done as indicated in the figures, and cell supernatants titrated on VeroE6 by plaque assay.

### Statistical analyses

T tests and ANOVA were done using Graphpad Prism. Dose–response analyses were by a generalized linear mixed model of the gamma family with log link function using a group-specific intercept and a common slope coefficient over the logarithm of the IFN-alpha concentration including intercept and slope as random factors to account for correlation within individual experiments (*P*-values adjusted for multiple testing). The analysis was performed in R 4.1.1 with glmer (lme4 1.1), Tukey-contrasts between the intercepts of the groups were determined using glht (multcomp 1.4–17) (17, 18).

## Data Availability

All data are included in the manuscript.

## References

[1] Viana R , et al. 2021. Rapid epidemic expansion of the SARS-CoV-2 Omicron variant in southern Africa. Nature. 603:679–686.. doi: 10.1101/2021.12.19.21268028. 10.1038/s41586-022-04411-y PMC8942855 35042229

[2] Grabowski F , Kochańczyk M, Lipniacki T. 2022. The spread of SARS-CoV-2 variant Omicron with a doubling time of 2.0–3.3 days can be explained by immune evasion. Viruses. doi: 10.3390/v14020294. 10.3390/v14020294 PMC8875689 35215887

[3] Garcia-Sastre A . 2017. Ten strategies of interferon evasion by viruses. Cell Host Microbe. 22:176–184. 28799903 10.1016/j.chom.2017.07.012 PMC5576560

[4] Palermo E , Di Carlo D, Sgarbanti M, Hiscott J. 2021. Type I interferons in COVID-19 pathogenesis. Biology. 10:829. 34571706 10.3390/biology10090829 PMC8468334

[5] Banerjee A , et al. 2021. Experimental and natural evidence of SARS-CoV-2-infection-induced activation of type I interferon responses. iScience. 24:102477. 33937724 10.1016/j.isci.2021.102477 PMC8074517

[6] Felgenhauer U , et al. 2020. Inhibition of SARS-CoV-2 by type I and type III interferons. J Biol Chem. 295:13958–13964. 32587093 10.1074/jbc.AC120.013788 PMC7549028

[7] Lokugamage KG , et al. 2020. Type I interferon susceptibility distinguishes SARS-CoV-2 from SARS-CoV. J Virol. 94. doi:10.1128/JVI.01410-20. 10.1128/JVI.01410-20 PMC7654262 32938761

[8] Holzer M , et al. 2019. Virus- and interferon alpha-induced transcriptomes of cells from the microbat *Myotis daubentonii*. iScience. 19:647–661. 31465999 10.1016/j.isci.2019.08.016 PMC6718828

[9] Peacock TP , et al. 2022. The altered entry pathway and antigenic distance of the SARS-CoV-2 Omicron variant map to separate domains of spike protein. bioRxiv. 10.1101/2021.12.31.474653

[10] Wang N , et al. 2020. Retrospective multicenter cohort study shows early interferon therapy is associated with favorable clinical responses in COVID-19 patients. Cell Host Microbe. 28:455–464e2. 32707096 10.1016/j.chom.2020.07.005 PMC7368656

[11] Loske J , et al. 2021. Pre-activated antiviral innate immunity in the upper airways controls early SARS-CoV-2 infection in children. Nat Biotechnol. 40:319–324.. doi: 10.1038/s41587-021-01037-9. 34408314 10.1038/s41587-021-01037-9

[12] Bastard P , et al. 2021. Autoantibodies neutralizing type I IFNs are present in ∼4% of uninfected individuals over 70 years old and account for ∼20% of COVID-19 deaths. Sci Immunol. 6:eabl4340. 34413139 10.1126/sciimmunol.abl4340 PMC8521484

[13] Jung C , et al. 2022. Omicron: what makes the latest SARS-CoV-2 variant of concern so concerning?. J Virol. 96:e0207721. 35225672 10.1128/jvi.02077-21 PMC8941872

[14] Rothe C , et al. 2020. Transmission of 2019-nCoV infection from an asymptomatic contact in Germany. N Engl J Med. 382:970–971. 32003551 10.1056/NEJMc2001468 PMC7120970

[15] Wilhelm A , et al. 2021. Antibody-mediated neutralization of authentic SARS-CoV-2 B.1.617 variants harboring L452R and T478K/E484Q. Viruses. 13:1693. 34578275 10.3390/v13091693 PMC8473269

[16] Wilhelm A , et al. 2021. Reduced neutralization of SARS-CoV-2 Omicron variant by vaccine sera and monoclonal antibodies. medRxiv. doi: 10.1101/2021.12.07.21267432.

[17] Bates D , Machler M, Bolker BM, Walker SC. 2015. Fitting linear mixed-effects models using lme4. J Stat Softw. 67:1–48.

[18] Hothorn T , Bretz F, Westfall P. 2008. Simultaneous inference in general parametric models. Biometrical J. 50:346–363. 10.1002/bimj.200810425 18481363

